# Hair Cortisol Concentrations as a Biological Marker of Maternal Prenatal Stress: A Systematic Review

**DOI:** 10.3390/ijerph17114002

**Published:** 2020-06-04

**Authors:** Mi-Young Kim, Go-Un Kim, Hae-Kyoung Son

**Affiliations:** 1 College of Nursing, Woosuk University, 443 Samnye-ro, Samnye-eup, Wanju-Gun, Jeollabuk-do 55338, Korea; miyoungkim726@gmail.com; 2 College of Nursing, Yonsei University, 50-1 Yonsei-ro, Seodaemun-gu, Seoul 03722, Korea; kgudfc@naver.com; 3 College of Nursing, Eulji University, 553 Sanseong-daero, Sujeong-gu, Seongnam-si 13135, Gyeonggi-do, Korea

**Keywords:** cortisol, hair, pregnancy, stress

## Abstract

Recently, biological markers of maternal prenatal stress, hair cortisol, along with saliva, blood, and urine cortisol, have received attention. However, it is necessary to validate measuring hair cortisol concentrations (HCC) as a biomarker of perceived stress among healthy and high-risk pregnant women. This study aimed to confirm the correlation between HCC and the perceived stress of pregnant women over 18 years of age. In this systematic review, we used various search engines to extract relevant articles using specific keywords related to pregnancy, hair cortisol, and psychological stress. Four out of 3639 studies met the inclusion criteria. We conducted a quality assessment with the help of three independent reviewers using the Strengthening the Reporting of Observational Studies in Epidemiology (STROBE) statement. The correlation between HCC and perceived stress was confirmed in one study. There was only one study on hair washing, shampoo, conditioner, and hair structure that could affect hair samples. In four studies, hair samples differed in length, methods of storage, and laboratory analysis. The review was limited to confirming the relationship between HCC and perceived stress in pregnant women based on the current evidence. Studies on hair cortisol need regulated and standardized methods for collection, storage, and analysis of hair samples.

## 1. Introduction

Stress induces the production of adrenal steroid hormones in pregnant women by increasing the secretion of the adrenocorticotropic hormone (ACTH), which may result in a high-risk pregnancy and complications such as preterm labor [1,2]. Moreover, an increase in the release of catecholamines due to the activation of the sympathetic nervous system, which is part of the autonomic nervous system, triggers physiological responses, such as contractions of the peripheral blood vessels and an increase in blood pressure and heart rate [3,4], which in turn adversely affects the blood flow of the fetus.

Preterm birth, defined as delivery before 37 weeks of gestation, is an important complication of both singleton and multifetal pregnancies worldwide [5,6]. More than 60% of all preterm births occur in Sub-Saharan Africa and South (-East) Asia [6]. Preterm birth with decreasing gestational age increases neonatal mortality and morbidity, and negatively affects maternal health and the family’s quality of life [7,8]. Medical interventions to reduce adverse pregnancy and birth outcomes that come with high-risk pregnancy, such as preterm birth, and to promote healthy birth for pregnant women, are needed.

In addition, as the social understanding of the importance of mother and child’s health increases worldwide, attention has been shifted, not only to maternal as physical health during pregnancy, but also psychological health. In particular, maternal prenatal stress is a significant factor affecting the general health of the pregnant woman and her fetus. Maternal prenatal stress has been associated with increasing cortisol levels, which may cause adverse pregnancy and birth outcomes [9]. 

Cortisol is a glucocorticoid hormone produced by the adrenal glands and increases when a person is subjected to stress [10]. Empirical literature revealed that the Perceived Stress Scale (PSS), which is a psychological instrument, and cortisol levels, are the most widely utilized measures of maternal stress during pregnancy [11]. Recently, hair cortisol concentration (HCC), as a biological marker of stress, has also received attention, along with saliva, blood, and urine cortisol [12,13]. While saliva, blood, and urine cortisol levels are appropriate for representing acute stress, HCC represents chronic stress [11]. Several studies have confirmed the utility of HCC as a biomarker of chronic stress [11,14,15]. In addition, an association has been reported between HCC and fetal gestational age [16,17].

Research based on the analysis of cortisol in hair samples began about a decade ago after the detection of several synthetic glucocorticoids in human hair [18,19]. Hair analysis is being increasingly and widely used to reflect exposure to drug abuse and environmental toxins including hair dye [20]. HCC can be analyzed by collecting hair samples from the posterior vertex region as close to the scalp as possible [21]. The non-invasive nature of hair sampling and easy storage of hair samples at room temperature further highlight the utility of this method for field research [22,23]. Due to diurnal variations, other samples either need to be taken at specific times of the day (i.e., saliva and serum) or require a laborious collection method (i.e., 24-h urine collection), making them unsuitable for population analysis [24]. Thus, compared with saliva, blood, or urine cortisol, hair cortisol can be easily obtainable for measuring long-term cortisol levels as a marker of chronic stress. Systematic review studies considering and verifying the validity of HCC as a biological marker of stress are still lacking compared to studies of saliva, blood, or urine cortisol. Therefore, this study systematically considers studies that utilized HCC as a biological marker of maternal prenatal stress and confirm the correlation between HCC and the perceived stress of pregnancy. 

## 2. Materials and Methods 

This study is a systematic review of the literature to compare HCC as a biomarker of stress and self-report measures of perceived stress among pregnant women.

### 2.1. Inclusion Criteria

To be eligible for the review, the following inclusion criteria were used:

(1) The study was performed on pregnant women over 18 years of age.
(2) Primary outcomes of the studies were HCC and perceived stress.
(3) The article was published in English but could be from any country.

### 2.2. Exclusion Criteria

To be eligible for the review, the following exclusion criteria were used:

(1) Pregnant women under the age of 18.
(2) Primary outcomes of the studies were saliva, serum, and urine cortisol.
(3) Studies using hair samples obtained from animal, not human participants.

### 2.3. Search Strategies

A systematic literature search was conducted in PubMed, Exceptra Medica data BASE (EMBASE), Cumulative Index to Nursing and Allied Health Literature (CINAHL), Cochrane Library, Research Information Sharing Service System (RISS), Korean Studies Information Service System (KISS), and National Digital Science Library (NDSL). For the grey literature search, we manually searched the Grey literature Report, a data base of clinical trials in the United States and Korea. Additionally, we searched the reference lists by hand.

We used the following search strings combining the relevant medical subject headings (MeSH) with additional keywords: (1) pregnancy, high-risk pregnancy, unwanted, pregnancy, unplanned pregnancy, twin pregnancy, triplet pregnancy, quadruplet pregnancy, quintuplet pregnancy, pregnant, gravidity; (2) hair cortisol, cortisol, hydrocortisone; and (3) psychological stress, physiological stress, stress. The search terms of this study were confirmed by librarians who specialize in reviewing the literature (Appendix A).

Eligibility was assessed independently in an unblended standardized manner by three team members and results were then compared. Disagreements between reviewers were discussed until consensus was reached.

### 2.4. Data Extraction and Quality Assessment

Data were extracted based on country, setting, study design, sample size, and general characteristics including age and mean gestational age. The results of HCC and perceived stress were independently extracted by three review authors and disagreements were discussed until a consensus was reached. We confirmed the sampling methods of hair (i.e., length and storage of hair samples, etc.) and laboratory analysis methods for HCC.

The quality of studies was evaluated by using the Strengthening the Reporting of Observational Studies in Epidemiology (STROBE) statement [25]. The score of each study ranged from 0 to 22 points. Scores of 19 points and over were considered as having very high quality, 15–18 points as high quality, 11–14 points as moderate quality, and 10 points or less as low quality. The three authors independently evaluated the selected studies. Disagreements between the authors were discussed until a consensus was reached. 

### 2.5. Data Analysis

We checked for HCC and perceived stress in the final selected studies, and the result values were compared and analyzed using Excel 2016, in accordance with the pregnancy trimester. There are limits to analysis in case measurement tools and units of measurement were not identical; therefore, we analyzed studies that allowed a quantitative synthesis of the corresponding result indicators.

### 2.6. Ethical Considerations

This study was exempted from Institutional Review Board (IRB) review by the Ethical Review Committee of Woosuk University (Approval no. WS-2019-3).

## 3. Results

### 3.1. Literature Search

A systematic review of the existing literature was performed to evaluate how well hair cortisol reflects perceived stress by comparing HCC and perceived stress in pregnant women. We retrieved a total of 3639 publications from the databases. Specifically, 1364 cases from PubMed, 1878 cases from Exceptra Medica data BASE (EMBASE), 270 cases from Cumulative Index to Nursing and Allied Health Literature (CINAHL), and 126 cases from Cochrane Library were found in the international database. One case from Research Information Sharing Service System (RISS), zero cases Korean Studies Information Service System (KISS), and zero cases from National Digital Science Library (NDSL) were found in the Korean databases. In the grey literature and its associated references, relevant articles were not available. Subsequently, 2309 of the retrieved articles were selected after excluding 1330 duplicate articles. In the primary selection process with a focus on the titles and abstracts, 62 of the 2309 articles met the inclusion criteria. After reviewing the remaining articles in full, four studies were finally selected. 

**Figure 1:**
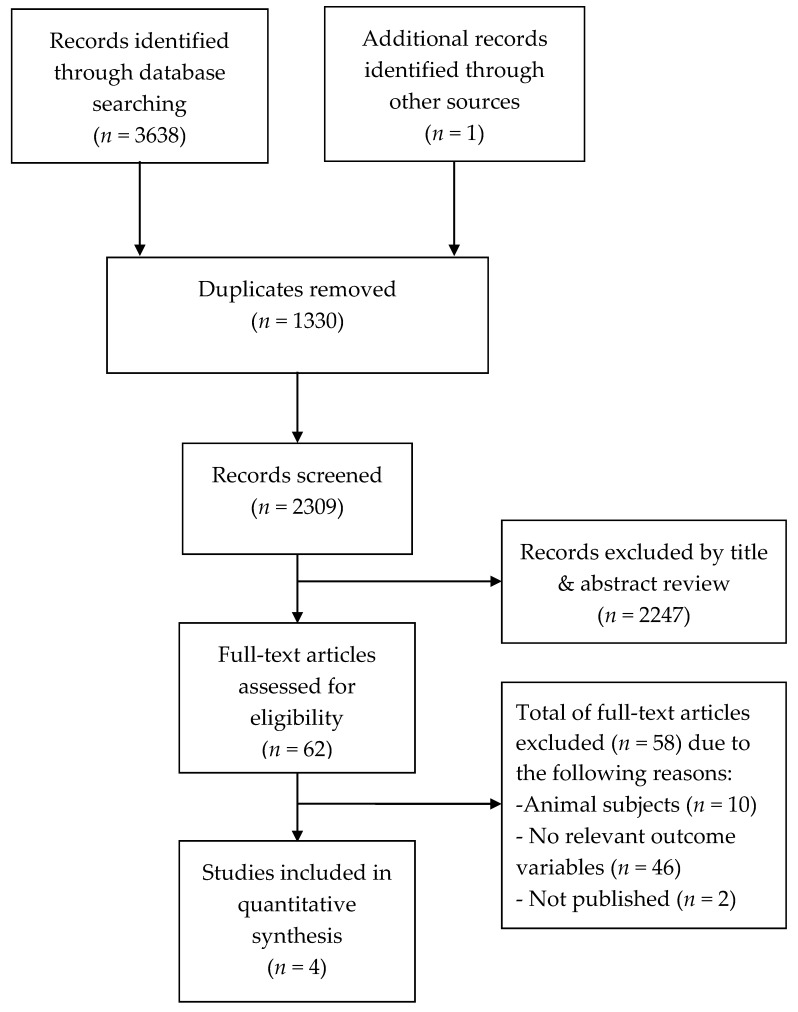
Flow chart of the results of the literature search.

The reasons for excluding the studies were as follows:

(1) Ten studies were conducted on animals (i.e., sheep, horses), not human participants;
(2) Forty-six studies in which the outcome variables were not HCC and perceived stress;
(3) Two studies were published at conferences, not in scientific journals (Figure 1).

### 3.2. General Characteristics of the Studies

The general characteristics of the final four selected studies are shown in Table 1. All of the four international studies were published in academic journals within the last 10 years as one study (25.0%) in 2007, one study (25.0%) in 2009, and two studies (50.0%) in 2018. The number of study participants was at least 25 to a maximum of 5337, and the average range of gestational age was from 13.1 to 37 weeks. One of four studies described the hair characteristics, including hair structure (i.e., dye, straighten, or perm, etc.) and hair products (i.e., hair washing, shampoo, conditioner, etc.). Three studies reported participants’ ethnicity. Two studies reported whether participants smoked or not.

### 3.3. Assessment of Methodological Quality

To assess the methodological quality of the selected studies, the study used the Strengthening the Reporting of Observational Studies in Epidemiology (STROBE) statement to assess the strengths and weaknesses of the investigation [25]. The STROBE statement includes items related to the title and abstract, introduction (i.e., background/rationale, etc.), methods (i.e., study design, setting, participants, variables, data sources/measurement, bias, etc.), results (i.e., descriptive data, outcome data, main results, etc.), discussion (i.e., limitation, interpretation, etc.), and other information (i.e., funding) sections of studies. The title and abstract in all studies were clear and the introduction presented the theoretical background and rationale. The methods presented the design including key concepts, setting, participants, and data collection. These also explained the variables to be investigated and confirmed the number of questionnaire items and the possible range of scores. However, one study did not clearly define the efforts to control potential confounders that threaten internal validity. In addition, it was not possible to ascertain how the sample size was calculated in all the papers, which simply reported the number of participants. Two studies described how missing data were addressed and clearly described the statistical analysis methods according to the objectives of the study. All studies described the number of participants included in the final analysis and explained the reasons for elimination. In addition, descriptive data related to the demographic characteristics of participants and outcome data could be found in all studies. In the discussion, the key results with references were summarized in all studies; however, only one study did not discuss its limitations. The discussion in all studies gave a cautious overall interpretation of results. In addition, the source of funding was clearly stated in all studies. Thus, the assessment of methodological quality by STROBE evaluated the selected studies as very high quality, with 21 points for Orta et al.’s [21] study, 21 points for Duffy et al.’s [26] study, 21 points for Kramer et al.’s [17] study, and 19 points for Kalra et al.’s [11] study.

### 3.4. Perceived Stress

In all studies, the level of perceived stress was evaluated by the Perceived Stress Scale (PSS) [27]. This scale is a self-report instrument that evaluates the level of perceived stress during the last month and consists of 14 items with a five-point response scale that consists of 0 points (never) to four points (very often). While two studies used the original version of the scale, the other two studies used a short 10-item version (PSS-10). The mean of perceived stress showed a distribution of 25.91 points at preterm, and from 20.4 points to 29.0 points at full term (Table 2).

### 3.5. Hair Cortisol Concentrations

The length of the hair samples varied from 3 to 10 cm. In two studies, sample sites were cut as close to the scalp as possible. The storage method of hair samples in the one study followed the guidelines of Duffy et al.’s [26] study. A piece of paper with the root and tip ends of hair sample was labeled and placed in Ziplock bags; this showed that the storage method of samples was used. Three studies suggested using methanol, but the amount and time of application were different. Two studies used a nitrogen stream to dry the hair samples, and only one study suggested buffering with presenting pH (Table 3).

**Figure 2:**
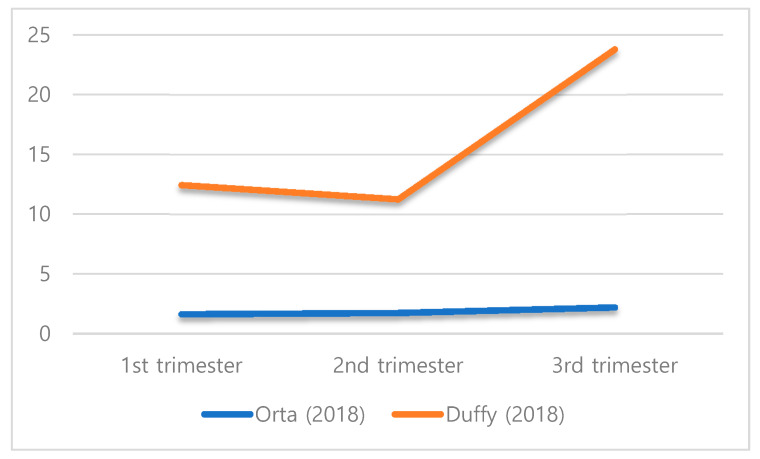
Variation of hair cortisol concentrations per trimester of pregnancy.

In the study by Orta et al. [21], HCC showed an increase from 1.64 in the first trimester to 2.22 in the third trimester of pregnancy. In the study by Duffy et al. [26], HCC also showed an increase from 12.45 to 23.80 during overall pregnancy; however, in the case of women with preterm labor, it showed a decrease from 9.71 in the first trimester to 8.59 in the third trimester (Figure 2).

## 4. Discussion

In this study, the final four studies were systematically considered to provide a fundamental basis for HCC as a biomarker of maternal prenatal stress. A systematic review of the literature showed an association between HCC and perceived stress among pregnant women. The results of Orta et al.’s [21] study found few associations between HCC and stress that were generally restricted to preconception and specific trimesters. In two studies, HCC from the first to the third trimester increased during the term of pregnancy, except for the second trimester in the study of Duffy et al. [26]. On the other hand, HCC in women who experienced preterm labor decreased as time passed in Duffy et al.’s [26] study. There was a lack of evidence to confirm the relationships between HCC and perceived stress in pregnant women who experienced preterm labor. In the case of high-risk pregnancies with preterm labor, pregnancy-related anxiety decreased as time passed because of the approach of the expected date of delivery (EDD). Perceived stress and pregnancy-related anxiety relating to poor pregnancy outcomes are known to be closely related and have a significant adverse impact on pregnant women and the fetus. In Dole et al.’s [28] study, a high level of pregnancy-related anxiety in pregnant women acted as a significant stressor that increased the spontaneous preterm birth rate. Maternal prenatal stress is also an important determinant of health among pregnant women and the fetuses. Given its potential effects during pregnancy, the American College of Obstetricians and Gynecologists [29] advocates for screenings of stress-related risk factors at least once per trimester followed by appropriated referrals. However, perceived stress in two studies was measured by the PSS [27], and the remaining studies used the short form of the scale (PSS-10). Thus, a cautious interpretation is required, considering the limitations, when comparing the results of perceived stress, as PSS only reflects the subject’s subjective stress level, and further investigations into its association with HCC from preconception to the third trimester should clarify this issue.

Participants of all four studies were pregnant women over 18 years of age covering a wide range of gestational periods from preconception to the third trimester of pregnancy; therefore, age and gender were not considered as confounders in the present study. However, in the three studies that specified the general characteristics of the pregnant woman, the participants had diverse characteristics, such as ethnicity (i.e., Mestizo, White, Black or African American, or Asian, etc.), marital status (i.e., legally married, cohabiting, living alone). Therefore, the comparison of the results is limited because of the lack of evidence for associations with HCC and sociodemographic variables as potential confounders in previous studies.

Moreover, Orta et al.’s [21] study investigated participants’ hair characteristics, including natural hair color (i.e., black or brown), hair structure (i.e., straight or curly), hair washing (i.e., 1–2, 3-5, 6-7 times per week), hair products (i.e., shampoo, conditioner, or hair chemicals such as tint, dye, or perm), and ultraviolet light exposure at enrollment (i.e., low, intermediate, or high). However, Orta et al.’s [21] study did not state whether hair characteristics affected HCC or were controlled as potential confounders.

Several human studies have reported no influence of hair characteristics (i.e., hair color, waves or curls, and hair washing, etc.) on HCC [30,31]. The results of Dettenborn et al.’s [30] study revealed a decreased effect of natural hair color, oral contraceptive use, and smoking status on HCC, and no influence of the frequency of hair washing on proximal hair segments except for the third hair segment, indicating lower cortisol content (*p* = 0.008). On the other hand, Sauvé et al.’s [10] study revealed that hair dye affects the shaft of the hair. HCC were found to be significantly reduced by shampoo washes in non-human primates, namely rhesus monkeys [32]. Further studies need to determine if HCC as a biomarker of chronic stress is affected by interval after hair dyeing or by repeated exposure to hair products such as shampoo and conditioner in humans, especially pregnant women. 

The descriptions of hair sampling, storage, and analysis were not consistent in the four studie; however, the length of hair samples varied in the studies from 3–10 centimeters. Human hair grows approximately one centimeter per month and hair samples were segmented to reflect HCC in preconception and each trimester [10,21]. In two studies, sample sites were cut as close to the scalp as possible. According to Kirschbaum, Tietze, Skoluda, and Dettenborn’s [33] study, there was a strong monotonic decline in HCC from the segment closest to the scalp to the most distal hair segment (*p* < 0.0001). Due to a rapid decline in HCC in adults, it can be a valid reflection of increased cortisol production for a period of up to six months [33]. The storage method of hair samples was performed following the guidelines of Duffy et al.’s [26] study. A piece of paper with the root and tip ends was labeled and placed in Ziplock bags in one study, showing the storage method of the hair samples. Furthermore, three studies suggested using methanol, but the amount and application times were different. Nitrogen stream was used for drying in two studies, and only one study suggested buffering with presenting the pH. These findings showed inconsistent methods of hair sampling, storage, and analysis to measure HCC. 

In general, due to the retrospective nature and the extended detection window of hair sampling, HCC should not be affected by immediate situational characteristics [22]. In other words, HCC can provide an integrated assessment of chronic hypothalamic–pituitary–adrenocortical (HPA) axis activity over a period of months that is not subjected to the influences of circadian variation or the stress of sample collection [10,23]. Considering the nature of hair cortisol samples in previous studies, HCC can be considered a useful measurement tool in stress-related psychobiological research. However, the identification of potential confounders is still an important step in establishing hair cortisol analysis [22]. Additional validation of hair as a suitable matrix for assessing long-term changes in HPA activity comes from the demonstration that HCC is elevated after exposure to a stressor associated with elevated circulating cortisol concentrations [32]. Future studies should consider a wide range of hair characteristics and examine their association with maternal prenatal stress. Furthermore, studies on HCC require regulated and standardized methods for the collection, storage, and analysis of hair samples.

## 5. Conclusions

This study was a systematic review of the literature that compared HCC as a biomarker of stress and perceived stress as psychometric measures among pregnant women. In further studies, through a systematic review of the literature, the interpretation of HCC, considering participants’ varying age, fetal gestational age, and obstetric history, is necessary. In other words, it is recommended that standardized guidelines for HCC measurements should be established. Although HCC is a non-invasive and useful measurement compared to saliva, blood, or urine cortisol, the generalizability of its validity will be established by identifying the sensitivity and specificity of HCC that reflects perceived stress.

## Figures and Tables

**Table 1:** Detailed description of reviewed articles.

Reference	Country; Setting	Study Design	Sample Size	Participant Age (Mean ± SD)	Mean Gestational Age (Weeks)	General Characteristics		
Orta et al., 2018 [21]	Peru; prenatal clinics	Prospective cohort study	97	Aged 18 years or older (26.5 ± 5.8)	13.1±3.9	- Mestizo ethnicity: 85.6% - Married or living with a partner 79.4% - Smoking 14.4%, alcohol 22.7% - Hair characteristics: black 55.7%, brown 44.3% - Hair structure: straight 68.0%, Curly 32.0% - Hair washing: 1–2 5.2%, 3–5 78.4%, 6–7 16.5% (per week) - Shampoo only 34.0%, Shampoo and conditioner 66.0%, Chemical hair treatment (tint, dye, or perm) 39.2%		
Duffy et al., 2018 [26]	USA; two hospitals	Prospective, cross-sectional design	52 preterm: 22 term: 30	Aged 18 years or older (preterm: 27.7 ± 7.1) (term: 27.4 ± 5.2)	Preterm: 24–37 Term: 37	Variables	Preterm, mean (SD)	Term, mean (SD)
						Previous pregnancies	2.5 (1.4)	1.4 (0.7)
						Never married	10 (45.5)	10 (33.3)
						Married	10 (45.5)	18 (60.0)
						Domestic partnership	2 (9.1)	2 (6.7)
						White	12 (54.5)	20 (66.7)
						Black or African American	6 (27.3)	4 (13.3)
						Asian	0 (0)	1 (3.3)
Kramer et al., 2009 [17]	Canada: four maternity hospitals	prospective cohort and a nested case-control design	5337 - Term Controls: 4885 - Total Cases: 207	Aged 18 years or older	24–26	Variables	Term Controls	Total Cases
						Legally married	46.2	39.2
						Cohabiting	43.8	51.0
						Living alone	10.1	9.8
						Medical/obstetric risk	34.5	51.0
						Smoking	15.6	16.6
						Primiparity	58.2	59.4
Kalra et al., 2007 [11]	Canada; hospital for sick children	Pilot study	25	Aged 18–45	End of the first or beginning of the second trimester of pregnancy			

**Table 2:** Comparison of perceived stress and hair cortisol concentrations.

Variables; Tool; Results		Orta et al., 2018 [21]	Duffy et al., 2018 [26]	Kramer et al., 2009 [17]	Kalra et al., 2007 [11]
Stress	Tool	Perceived Stress Scale (PSS)	Perceived Stress Scale (PSS)	Perceived Stress Scale (PSS–10)	Perceived Stress Scale (PSS-10)
	Results	29.0 (4.9)	– Preterm 25.91 (7.0) (range 12–42) – Term 20.40 (6.3) (range 7–32)	– Term 4.0 (3.1), T – Total cases: 4.3 (3.0)	10.6 ± 5.81 (range 2–22)
Hair cortisol concentrations	Tool	– preconception: 3–6 cm – 1st trimester: 3 cm – 2nd trimester: 3–6 cm – 3rd trimester: 3 cm	– at least 10 cm, 30 mg	9 cm	- 1–1.5 cm - at least 10 mg
	Results	– preconception: 1.28 (1.00) ∙ 1st trimester: 1.64 (0.96) ∙ 2nd trimester: 1.75 (0.89) ∙ 3rd trimester: 2.22 (0.88)	– First trimester (pg/mg) ∙ preterm 9.71 (5.5) (range 0–123.7) ∙ term 12.45 (21.7) (range 0–82.4) – Second trimester ∙ preterm 8.71 (3.4) (range 0.7–78.4) ∙ term 11.26 (15.6) (range 0–74.2) – Third trimester ∙ preterm 8.59 (2.0) (range 0–41.9) ∙ term 23.80 (65.2) (range 0–357.1)	– Term 190.6 ng/g (99.0) – Total cases 171.7 ng/g (76.4)	0.133 ± 0.048, (range 0.064–0.234)
Correlation		Preconception-1st Trimester: 0.83nh * Preconception-2nd Trimester: 0.23 * Preconception-3rd Trimester: 0.15 1st Trimester-2nd Trimester: 0.19 1st Trimester-3rd Trimester: 0.08 2nd Trimester-3rd Trimester: 0.75 ***	3rd Trimester: t = 2.16, df = 48, *p* = 0.04 2nd Trimester: t = 1.88, df = 48, *p* = 0.06 over time between groups: F(2, 135) = 9.51, *p* ≤ 0.01,	–	Rs = 0.47, *p* < 0.05

* *p* < 0.05, *** *p* < 0.0001.

**Table 3:** Laboratory analysis of hair samples.

Reference		Hair Cortisol
Orta et al., 2018 [21]	hair samples	- preconception: 3–6 cm - 1st trimester: 3 cm - 2nd trimester: 3–6 cm - 3rd trimester: 3 cm
	laboratory analysis	- 2.5 mL isopropanol wash: three minutes each time - dry: 12 h - 1.8 ml high-grade methanol: 18 h - 55 °C using a steady stream of nitrogen: 30 min - resuspended in 225 microliters (mL) of distilled water - added 20 mL of internal standard (cortisol-d4)
Duffy et al., 2018 [26]	hair samples	- at least 10 cm, 30 mg - sample site: cut occurring as close to the scalp as possible - taped hair samples with the root and tip ends labeled - placed them in Ziplock bags - stored in an 80 °C freezer
	laboratory analysis	- grinded it for 6 min at 25 Hz - methanol overnight - dried under a nitrogen stream - Intra-assay coefficients of variation: at less than 10%
Kramer et al., 2009 [17]	hair samples	- hair closest to the scalp - double plastic bag - refrigerated
	laboratory analysis	- 3-mm thickness was cut from the fresh placenta - high intra-observer agreement (j ¼ 0.50–0.78)
Kalra et al., 2007 [11]	hair samples	- 1–1.5 cm - at least 10 mg
	laboratory analysis	- methanol 1 mL, sonicator: 45 min, 50 °C overnight - 1 mL Thermodyne, buffered at pH 7.2

## Appendix A

1. Search Terms

(1) PubMed: 1364

(((((((“Pregnancy”[Mesh]) OR (“Pregnancy, High-Risk”[Mesh] OR “Pregnancy, Unwanted”[Mesh] OR “Pregnancy, Unplanned”[Mesh] OR “Pregnancy, Twin”[Mesh] OR “Pregnancy, Triplet”[Mesh] OR “Pregnancy, Quadruplet”[Mesh] OR “Pregnancy, Quintuplet”[Mesh] OR “Pregnancy, Angular”[Mesh]))) OR ((Pregnancy OR Pregnancy, High-Risk OR Pregnancy, Unwanted OR Pregnancy, Unplanned OR Pregnancy, Twin OR Pregnancy, Triplet OR Pregnancy, Quadruplet OR Pregnancy, Quintuplet OR Pregnancy, Angular))) OR ((pregnant or gravidity)))) AND (((((hair cortisol or cortisol))) OR "Hydrocortisone"[Mesh])) AND (((("Stress, Psychological"[Mesh] OR "Stress, Physiological"[Mesh])) OR ((Stress, Psychological OR Stress, Physiological))) OR stress)

(2) EMBASE: 1878

(‘pregnancy’/exp OR ‘high risk pregnancy’/exp OR ‘unwanted pregnancy’/exp OR ‘unplanned pregnancy’/exp OR ‘twin pregnancy’/exp OR ‘triplet pregnancy’/exp OR ‘quadruplet pregnancy’/exp OR ‘quintuplet pregnancy’/exp OR ‘angular pregnancy’/exp OR pregnancy OR ‘high risk pregnancy’ OR ‘unwanted pregnancy’ OR ‘unplanned pregnancy’ OR ‘twin pregnancy’ OR ‘triplet pregnancy’ OR ‘quadruplet pregnancy’ OR ‘quintuplet pregnancy’ OR ‘angular pregnancy’ OR pregnant OR gravidity) AND (‘hydrocortisone’/exp OR ‘hair cortisol’ OR cortisol) AND (‘stress’/exp OR ‘mental stress’/exp OR ‘physiological stress’/exp OR ‘psychological stress’ OR ‘physiological stress’ OR stress OR ‘mental stress’)

(3) CINAHL: 270

((MH “Pregnancy”) OR (MH “Pregnancy, High Risk”) OR (MH “Pregnancy, Unwanted”) OR (MH “Pregnancy, Unplanned”) OR (MH “Pregnancy, Twin”) OR (MH “Pregnancy, Triplet”) OR (MH “Pregnancy, Quadruplet”) OR (MH “Pregnancy, Quintuplet”) OR ((Pregnancy OR Pregnancy, High-Risk OR Pregnancy, Unwanted OR Pregnancy, Unplanned OR Pregnancy, Twin OR Pregnancy, Triplet OR Pregnancy, Quadruplet OR Pregnancy, Quintuplet OR Pregnancy, Angular))) OR ((pregnant or gravidity))) AND ((MH "Hydrocortisone") OR (hair cortisol or cortisol)) AND (((MH “Stress”) OR (MH “Stress, Physiological”) OR (MH “Stress, Psychological”)) OR (Stress, Psychological OR Stress, Physiological OR stress))

(4) Cochrane Library: 126

([Pregnancy] explode all trees OR [Pregnancy, High-Risk] explode all trees OR [Pregnancy, Unwanted] explode all trees OR [Pregnancy, Unplanned] explode all trees OR [Pregnancy, Twin] explode all trees OR [Pregnancy, Triplet] explode all trees OR [Pregnancy, Quadruplet] explode all trees OR [Pregnancy, Quintuplet] explode all trees OR [Pregnancy, Quintuplet] explode all trees OR Pregnancy OR Pregnancy, High-Risk OR Pregnancy, Unwanted OR Pregnancy, Unplanned OR Pregnancy, Twin OR Pregnancy, Triplet OR Pregnancy, Quadruplet OR Pregnancy, Quintuplet OR Pregnancy, Angular OR pregnant OR gravidity) AND ([Hydrocortisone] explode all trees OR hair cortisol OR cortisol) AND ([Stress, Psychological] explode all trees OR [Stress, Physiological] explode all trees OR Stress, Psychological OR Stress, Physiological OR stress)

(5) RISS, KISS, NDSL, Grey literature: 1

Pregnancy AND Cortisol AND Stress
